# Endotoxemia-Induced Release of Pro-inflammatory Mediators Are Associated With Increased Glomerular Filtration Rate in Humans *in vivo*

**DOI:** 10.3389/fmed.2020.559671

**Published:** 2020-11-05

**Authors:** Remi Beunders, Maren J. Schütz, Roger van Groenendael, Guus P. Leijte, Matthijs Kox, Lucas T. van Eijk, Peter Pickkers

**Affiliations:** ^1^Department of Intensive Care Medicine, Radboud University Medical Center, Nijmegen, Netherlands; ^2^Radboud Center for Infectious Diseases (RCI), Radboud University Medical Center, Nijmegen, Netherlands; ^3^Radboud Institute for Molecular Life Sciences, Radboud University Medical Center, Nijmegen, Netherlands; ^4^Department of Anesthesiology, Pain and Palliative Medicine, Radboud University Medical Center, Nijmegen, Netherlands

**Keywords:** augmented renal clearance, sepsis, acute kidney injury, systemic inflammation, iohexol plasma clearance, glomerular filtration rate, endogenous creatinine clearance

## Abstract

**Introduction:** Sepsis is the most prevalent cause of Acute Kidney Injury (AKI). Conversely, in some septic patients the glomerular filtration rate (GFR) is augmented. The role of the inflammatory response and blood pressure to induce this increased GFR is unknown. Herein, we relate inflammatory mediators and blood pressure to the iohexol clearance-derived “true” GFR and kidney injury markers during systemic inflammation in healthy volunteers.

**Methods:** Twelve healthy subjects underwent experimental endotoxemia (i.v. administration of 2 ng/kg Escherichia coli-derived lipopolysaccharide, LPS). As a gold-standard to determine the GFR, iohexol plasma clearance (GFR_iohexol_) was calculated during a 6-h period on the day before (baseline) as well as 2 and 24 h after LPS administration. Intra-arterial blood pressure was recorded continuously using a radial artery catheter. Circulating inflammatory mediators and urinary excretion of kidney injury markers were serially measured.

**Results:** Experimental endotoxemia profoundly increased plasma concentrations of inflammatory mediators, including [mean ± SD or median [IQR] peak values (pg/mL) of tumor necrosis factor (TNF)-α: 92 ± 40, interleukin (IL)-6: 1,246 ± 605, IL-8: 374 ± 120, IL-10: 222 ± 119, IL-1 receptor antagonist (RA): 8,955 ± 2,429, macrophage chemoattractant protein (MCP)-1: 2,885 [2,706 – 3,765], vascular adhesion molecule (VCAM)-1: 296,105 ± 34,822, intercellular adhesion molecule (ICAM)-1: 25,0170 ± 41,764]. Mean arterial pressure decreased with 13 ± 11 mmHg (*p* < 0.0001). No significant increase in the urinary excretion of tubular injury markers was observed following LPS administration. GFR_iohexol_ increased from 97 ± 6 at baseline to 118 ± 10 mL/min/1.73m^2^ (*p* < 0.0001) post-LPS administration and returned to baseline levels at 24 h post-LPS (99 ± 9 mL/min/1.73m^2^). Peak plasma concentrations of IL-6 (*R*^2^ = 0.66, *p* = 0.001) and IL-8 (*R*^2^ = 0.51, *p* = 0.009), MCP-1 (*R*^2^ = 0.38, *p* = 0.03) and VCAM-1 levels (*R*^2^ = 0.37, *p* = 0.04) correlated with the increase in GFR_iohexol_, whereas a trend was observed for TNF-α (*R*^2^ = 0.33, *p* = 0.0509) and IL-1RA (*R*^2^ = 0.28, *p* = 0.08). None of the kidney injury markers or changes in blood pressure were associated with GFR_iohexol._ In multiple linear regression analysis, both peak IL-6 (*p* = 0.002) and IL-8 (*p* = 0.01) concentrations remained significantly correlated with GFR_iohexol_, without collinearity.

**Discussion:** Concentrations of pro-inflammatory cytokines, but not blood pressure, are correlated with the endotoxemia-induced increase in GFR in healthy volunteers. These findings may indicate that inflammatory mediators orchestrate the augmented GFR observed in a subgroup of sepsis patients.

## Introduction

Sepsis influences renal function. Naturally, most of the focus is on sepsis-associated deterioration of renal function leading to Acute Kidney Injury (AKI) in sepsis patients. Sepsis is the most commonly observed cause of AKI, and AKI is often severe in this group of patients (1). The inflammatory environment in the kidney may lead to the redistribution of intrarenal perfusion (2) and subsequent deterioration of the renal microcirculation.

On the other hand, renal hyperfiltration, defined as increased creatinine clearance ≥130 mL/min/1.73m^2^, is also observed in sepsis patients (3), with a reported prevalence ranging from 40 to 65% (4–6). The high cardiac output often observed in the early phase of sepsis (7, 8), appears to be the most important predictor of increased renal blood flow (7). Furthermore, renal functional reserve is probably necessary for an increase in RBF to induce an increased GFR (4). Importantly, while augmented renal clearance may represent the renal reserve and is the opposite of AKI, it may have detrimental clinical consequences, for instance due to influencing the plasma concentrations of renally excreted agents, such as antibiotics. Accordingly, evidence suggests that renal hyperfiltration is associated with impaired outcome in the general ICU population receiving antibiotic treatment (9). Yet, the mechanisms driving renal clearance in the critically ill remain poorly understood.

The aim of this study was to investigate the relation between systemic inflammation, blood pressure, and kidney function in healthy volunteers challenged with intravenous administered bacterial endotoxin.

## Materials and Methods

### Study Population

Data of healthy volunteers randomized to the placebo group of a previously performed study (10) were used for the analyses described in the present work. The study was approved by the ethics committee CMO Arnhem-Nijmegen (NL56102.091.15; 2015-2231), registered at clinicaltrials.gov (NCT02629874) and conducted according to the ethical principles of the Declaration of Helsinki ICH E6 (R1), the Dutch Medical Research Involving Human Subjects Act and the guidelines of Good Clinical Practice. All subjects provided written informed consent. Quality assurance, monitoring and full data validation was performed by an independent contract research organization.

Healthy male volunteers with a minimum age of 18 years were screened for eligibility. Exclusion criteria consisted, among others, of a body mass index of <18 or >30 kg/m^2^, illness in the 2 weeks before start of the study and significant blood loss within 90 days prior to the study. The use of medication, recreational drugs, nicotine, caffeine, and alcohol were prohibited during the duration of the study.

### Study Procedures

**Figure 1:**
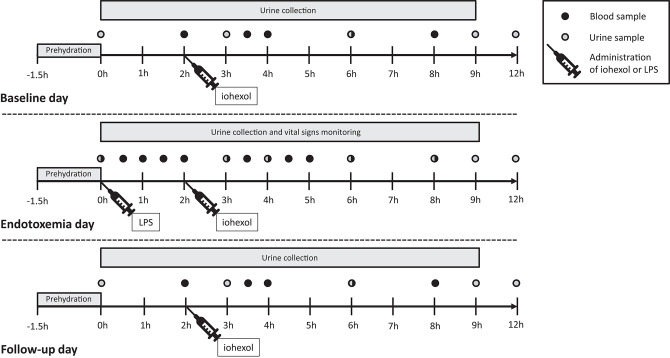
Study design. Twelve healthy male volunteers were hospitalized and underwent experimental endotoxemia (intravenous administration of 2 ng/kg *E. coli*-derived lipopolysaccharide). Inflammatory mediators, blood pressure, and glomerular filtration rate were measured at three consequent days: baseline day, endotoxemia day, and follow-up day. LPS, lipopolysaccharide; h, hours.

Study procedures were performed on 3 consecutive days on our intensive care unit: a baseline day, the day of the endotoxin challenge and a follow-up day. A complete overview of the study design is depicted in Figure 1. Escherichia coli-derived lipopolysaccharide (LPS, 2 ng/kg, purified [US Standard Reference Endotoxin Escherichia Coli O:113], [NIH, Bethesda, MD, USA]) was administered to induce a transient systemic inflammatory response. To pre-hydrate the subjects, 1.5 L 2.5% glucose/4.5% NaCl was administered intravenously in the hour before LPS administration, or at the same time point on the baseline and follow-up day, according to our standardized endotoxemia protocol (11). Following LPS administration, 250 mL/hour 2.5% glucose/ 0.45% NaCl was administered for 2 hours, followed by 150 mL/hour until discharge, 9 h after endotoxin challenge. Fluid administration was identical on the three study days. Cardiac rhythm was monitored using a 3-lead electrocardiogram and blood pressure was continuously measured using a 20-gauge arterial catheter. Data was recorded using a Philips MP50 patient monitor and an in-house developed data capturing system.

### Iohexol-Based GFR Measurements

Iohexol plasma clearance has a very strong correlation with inulin clearance: *r*^2^ = 0.96 (12). The water-soluble iodine contrast agent iohexol (OMNIPAQUE 240, containing 518 mg/mL iohexol and 240 mg iodine/mL, GE Healthcare, Eindhoven, the Netherlands) was administrated as a single intravenous bolus of 5 mL (13) at 2 h after LPS administration or at the same time point on the baseline and follow-up day, see Figure 1. The blood samples to determine iohexol concentration were obtained at 2, 3.5, 4, 6, and 8 h after LPS administration, centrifuged at 2,000 g for 10 min at 4° Celsius (C) and stored at −80°C until analysis using High Performance Liquid Chromatography (HPLC) at the department of Pharmacology and Toxicology, Radboudumc Nijmegen. The plasma disappearance curve of iohexol was used to calculate the GFR using the slope interception method, as described previously (13). The GFR was corrected using the Brøchner-Mortensen correction and for body surface area using the Mosteller formula (14). GFR_iohexol_ measurements were conducted on all 3 days (Figure 1).

### Creatinine-Based GFR Assessments

The GFR was also calculated using endogenous creatinine clearance (GFR_ECC_) on all 3 days. Urine was collected for a period of 9 h and plasma and urine was sampled at the end of the collection period to determine creatinine concentrations according to routine clinical laboratory analysis methods.

### Urinary Excretion of Kidney Injury Markers

For determination of kidney injury markers, urine was sampled on the endotoxemia day at 0, 3, 6, 9, and 12 hours following LPS administration or at the same time points on the baseline and follow-up day. Urine was homogenized and samples were stored at −80° C until analysis. Concentrations of neutrophil gelatinase-associated lipocalin (NGAL), and kidney injury molecule (KIM)-1 were measured using enzyme-linked immunosorbent assays (ELISAs, Duoset, R&D systems, McKinley, USA), as were levels of liver-type fatty acid binding protein (L-FABP, CMIC holdings, Tokyo, Japan). Levels were normalized for urinary creatinine concentrations.

### Circulating Inflammatory Mediators

Ethylenediaminetetraacetic acid (EDTA)-anticoagulated blood samples for measurement of inflammatory parameters were obtained at 0, 0.5, 1, 1.5, 2, 3, 4, 6, and 8 h following LPS administration. Samples were immediately centrifuged at 2,000 g for 10 min at 4°C after which plasma was stored at −80°C until analysis of tumor necrosis factor (TNF)-α, interleukin (IL)-6 and IL-10, IL-8, IL-12, IL-1 receptor antagonist (RA), monocyte chemoattractant protein (MCP)-1, macrophage inflammatory protein (MIP)1-α, MIP1-β, intercellular adhesion molecule (ICAM)-1, and vascular cell adhesion protein (VCAM)-1. Levels of IL-6, IL-10, and TNF-α were determined using a validated ISO9001 certified multiplex immunoassay (Luminex, Austin, TX, USA) at the Laboratory of Translational Immunology of the University Medical Center Utrecht, as described elsewhere (15), whereas concentrations of IL-8, MCP-1, IL-1RA, MIP-1α, MIP-1β, ICAM-1, and VCAM-1 were determined using a Luminex assay according to the manufacturer's instructions (ICAM-1 and VCAM-1: Bio-plex, Bio-rad, Hercules, CA, USA; rest: Milliplex; Merck Millipore, Billerica, MA, USA).

### Statistical Analysis

Data were tested for normality using the Shapiro-Wilk test and presented as mean ± standard deviation (mean ± standard error of the mean in figures), or median [interquartile range]. One-way repeated measures analysis of variance (ANOVA) was used to test serial data. Correlations were calculated using Pearson's correlation coefficient. Multiple linear regressions were performed and collinearity was evaluated. Logarithmic transformation was used if data was not normally distributed. A *p*-value of <0.05 was considered statistically significant. The data was analyzed with SPSS version 25 (IBM, Armonk, NY, USA), ANOVA analysis and figures were made using GraphPad Prism version 5.03 (GraphPad Software, La Jolla, CA, USA).

## Results

Twelve healthy male volunteers, aged 23 ± 3 years, were enrolled in the study. The demographic characteristics of the study population are listed in Table 1.

**Table 1:** Demographics of the study population.

	**Healthy male volunteer (*n* = 12)**
Age (years)	23 ± 3
Length (cm)	183 ± 5
Weight (kg)	77 ± 8
BMI (kg/m^2^)	23.2 ± 2.5
BSA (m^2^)	2.0 ± 0.1
MAP (mmHg)	95 ± 11
GFR_iohexol_ (mL/min/1.73m^2^)	97 ± 6
GFR_ECC_ (mL/min/1.73m^2^)	152 ± 16

*Data is presented as mean ± standard deviation. BMI, body mass index; BSA, body surface area; MAP, mean arterial pressure; GFR, glomerular filtration rate; ECC, endogenous creatinine clearance*.

### Systemic Inflammatory Response Parameters

**Figure 2:**
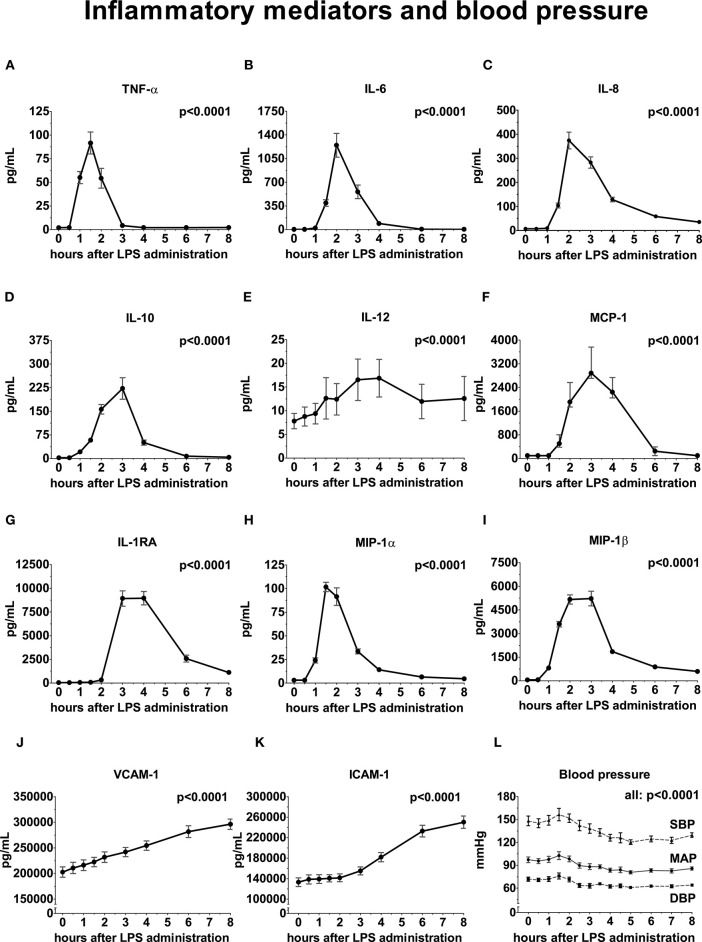
Inflammatory mediators and blood pressure following LPS administration. All variables are depicted over time, relative from LPS administration, starting from 0 h until 8 h. **(A)** Tumor Necrosis Factor-alpha (TNF-α), **(B)** Interleukin (IL)-6, **(C)** IL-8, **(D)** IL-10, **(E)** IL-12, **(F)** Monocyte Chemoattractant Protein-1, **(G)** IL-1 Receptor Antagonist, **(H)** Macrophage Inflammatory Protein-1α, **(I)** MIP-1β, **(J)** Vascular Cell Adhesion Molecule, **(K)** Intercellular Adhesion Molecule, **(L)** Blood pressure. Variance over time tested using a repeated measures one-way ANOVA. SBP, systolic blood pressure; MAP, mean arterial pressure; DBP, diastolic blood pressure.

Following LPS administration, a swift and profound increase of both pro- and anti-inflammatory cytokines, chemokines, and vascular adhesion molecules was observed, illustrated in Figure 2 (peak values [all in pg/mL] of TNF-α: 92 ± 40 *p* < 0.0001, IL-6: 1,246 ± 605 *p* < 0.0001, IL-8: 374 ± 120 *p* < 0.0001, IL-10: 222 ± 119 *p* < 0.0001, IL-12: 14.5 [9.3 – 20.6] *p* < 0.0001, IL-1RA: 8,955 ± 2,429 *p* < 0.0001, MCP-1: 2885 [2,706 – 3,765] *p* < 0.0001, MIP-1α: 102 ± 17 *p* < 0.0001 and MIP-1β: 5,162 ± 1,016 *p* < 0.0001, VCAM-1: 296,105 ± 34,822 *p* < 0.0001, and ICAM-1: 250,170 ± 41,764 *p* < 0.0001.

### Kidney Function and Urinary Excretion of Tubular Injury Markers

**Figure 3:**
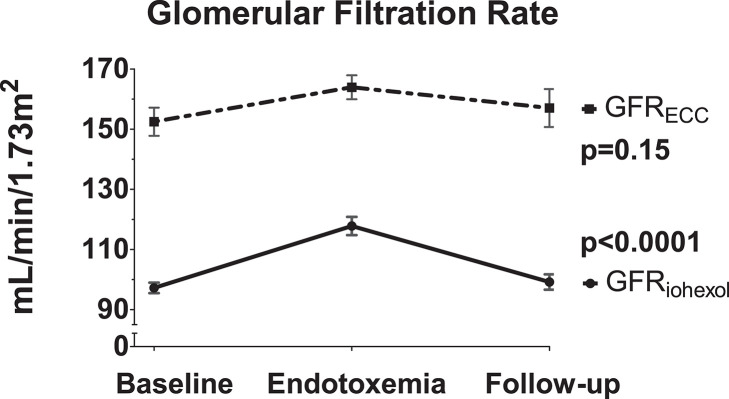
Kidney function changes due to LPS administration. The iohexol-derived GFR and GFR calculated using endogenous creatinine clearance are determined on the three consecutive study days: baseline day prior to LPS administration, endotoxemia day in the hours following LPS administration and the follow-up day after LPS administration. Variance over time tested using a repeated measures one-way ANOVA.

**Figure 4:**
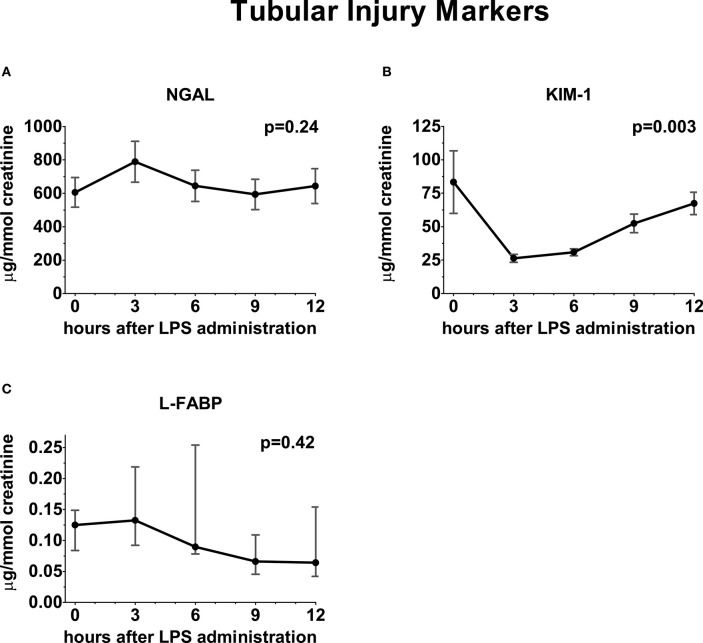
Urinary excretion of tubular injury markers following LPS administration. Concentration of tubular injury markers in urine depicted over time relative from LPS administration, starting from 0 h until 12 h. **(A)** Neutrophil gelatinase-associated lipocalin, **(B)** Kidney injury molecule-1, **(C)** Liver fatty acid-binding protein. Variance over time tested using a repeated measures one-way ANOVA.

Following administration of LPS, a significant increase in GFR_iohexol_ was observed, which returned to baseline levels on the follow-up day (baseline: 97 ± 6; endotoxemia day: 118 ± 10; follow-up day: 99 ± 9 ml/min/1.73m^2^, *p* < 0.001, Figure 3). GFR_ECC_ showed a similar trend, but changes did not reach statistical significance (baseline: 152 ± 16; endotoxemia day: 164 ± 14; follow-up day: 157 ± 22 mL/min/1.73m^2^, *p* = 0.15, Figure 3). LPS administration did not result in an increase of the urinary tubular injury markers NGAL and L-FABP, whereas urinary KIM-1 concentrations significantly decreased following LPS administration (*p* = 0.0025, Figure 4).

### Hemodynamic Parameters

The mean arterial pressure (MAP) initially increased post-LPS administration, peaking at 1.5 h (from 94 ± 10 to 103 ± 18 mmHg), followed by a decrease with a nadir at 5 h after LPS administration (−13 ± 11 mmHg compared to baseline, Figure 2). The systolic and diastolic blood pressure decreased by 22 ± 17 and 9 ± 9 mmHg compared with baseline, respectively (*p* = 0.0003 and *p* = 0.003) (Figure 2).

### Relationship Between Inflammatory Markers and Iohexol-Based GFR

**Figure 5:**
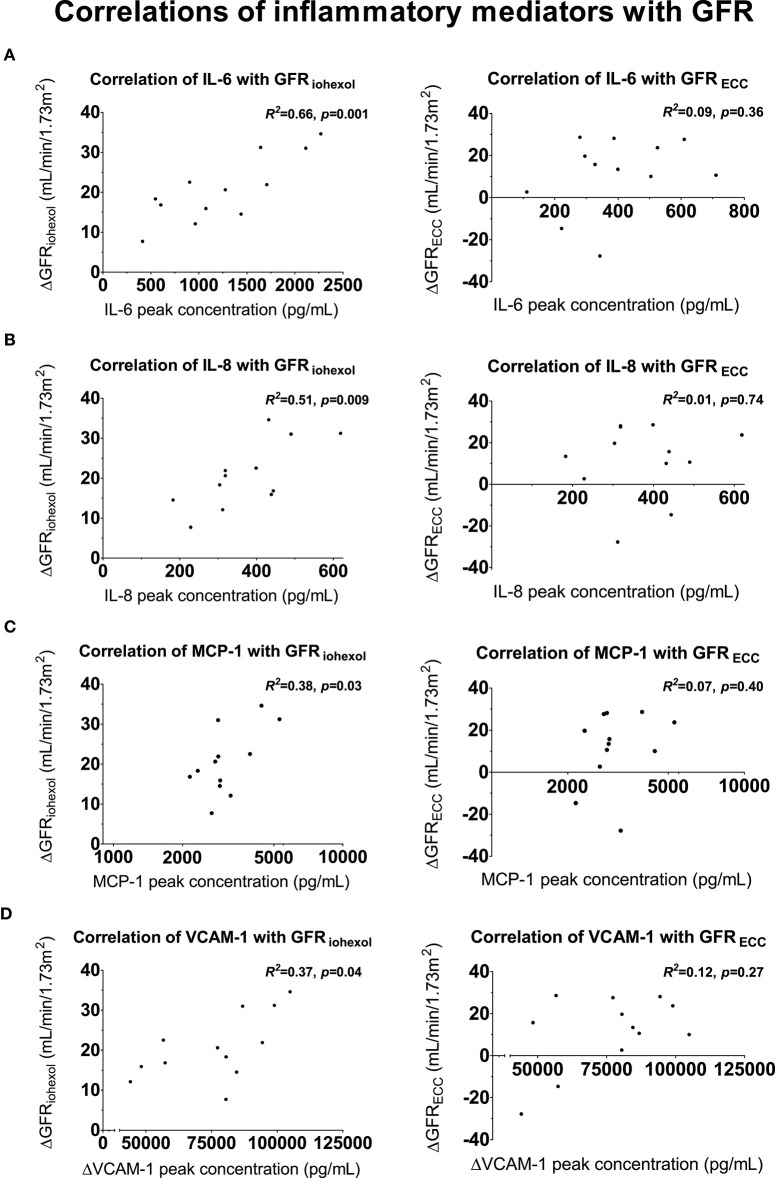
Correlations between inflammatory mediators and GFR. Scatter plots of peak concentrations of inflammatory mediators correlated with the increase in glomerular filtration rate (GFR, iohexol-derived and using endogenous creatinine clearance) on the endotoxemia day compared to the baseline day. **(A)** Proinflammatory cytokine interleukin (IL)-6 with GFR_iohexol_ (left) and GFR_ECC_ (right), **(B)** proinflammatory cytokine IL-8 with GFR_iohexol_ (left) and GFR_ECC_ (right), **(C)** Monocyte Chemoattractant Protein-1 with GFR_iohexol_ (left) and GFR_ECC_ (right), **(D)** Vascular Cell Adhesion Molecule-1 correlated with GFR_iohexol_ (left) and GFR_ECC_ (right). For a complete overview of all correlations with GFR_iohexol_ and GFR_ECC_, see Supplementary Table 1. Correlations were tested using Pearson's correlation coefficient.

Peak plasma concentrations of the pro-inflammatory cytokines IL-6 (*R*^2^ = 0.66, *p* = 0.001) and IL-8 (*R*^2^ = 0.51, *p* = 0.009), MCP-1 (*R*^2^ = 0.38, *p* = 0.03) and the maximum increase in VCAM-1 levels (*R*^2^ = 0.37, *p* = 0.04) were significant correlated with the increase in GFR_iohexol_ (Figure 5 and Supplementary Table 1), whereas trends were observed for TNF-α (*R*^2^ = 0.33, *p* = 0.0509) and IL-1RA (*R*^2^ = 0.28 *p* = 0.08). Peak levels of IL-10, IL-12, MIP-1α, MIP-1β, and ICAM-1 did not correlate with GFR_iohexol_. In a multiple linear regression analysis, with GFR_iohexol_ as dependent variable and the cytokines that correlated with GFR_iohexol_ as independent variables, peak IL-6 (*p* = 0.002) and IL-8 (*p* = 0.01) remained independently associated with the increase in GFR_iohexol_. Peak IL-6 and IL-8 levels showed no collinearity with regard to their association with GFR_iohexol_ (VIF = 1.185, VIF = 1.185). The peak plasma concentrations of the inflammatory mediators did not correlate with the non-significant change in GFR_ECC_ following LPS administration (Figure 5 and Supplementary Table 1).

### Relationship Between Blood Pressure and GFR

The peak and nadir of the arterial blood pressures (SBP, DBP, and MAP) following LPS administration did not correlate with the change in GFR_iohexol_ and GFR_ECC_ (Supplementary Table 2).

## Discussion

In this study in healthy volunteers with a systemic immune response elicited by endotoxin administration, increases in inflammatory mediators were significantly associated with the increase in the “true GFR” measured by plasma clearance of iohexol. This is the first report demonstrating a direct relationship between the systemic inflammatory response and increased GFR in humans.

Sepsis influences renal function. On the one hand AKI, defined as a decrease in GFR, clearly recognized in the clinic, but on the other hand also an augmented GFR is possible, a phenomenon that is much less known. Renal hyperfiltration is now increasingly being recognized as a clinical entity (16–18). A highly relevant clinical consequence is the augmented clearance of renally excreted drugs, most notably antibiotics, which is important for daily practice as well as for clinical trials investigating novel therapeutic compounds (19–21). Adjustment of the dosing of these drugs should be considered when GFR is increased, as is already common practice when the GFR is decreased (22). The increase in GFR during early sepsis has up to now mainly been explained from altered hemodynamics. The hyperdynamic circulation observed in these patients is characterized by a high cardiac output which is the most important and independent predictor of renal blood flow (RBF) (7). In addition, redistribution of blood flow through the kidney during systemic inflammation may result in hyperfiltration (4, 7). However, to the best of our knowledge, we here describe for the first time that the increase in GFR may also reflect a direct consequence of the inflammatory process, independent of hemodynamic changes.

Pro-inflammatory cytokines, such as TNF-α, IL-6, and IL-8, are important orchestrators of the innate immune response and are associated with impaired outcome in ICU patients (23, 24). Until now, many studies have focused on the occurrence of AKI and a decrease of GFR due to inflammation (25, 26). This is in contrast with the relation between inflammation and increase in GFR that we demonstrate. It is known that Damage- and Pathogen Associated Molecular Patterns (DAMPs and PAMPs) such as cytokines and LPS interact directly with tubular cells (27) and induce a pro-inflammatory cascade (28). A dysregulated microcirculation in the kidney during systemic inflammation may lead to a prolonged exposure of the inflammatory mediators in regions with low blood flow and sustain the inflammatory response and its consequences. It appears plausible that both the intensity as well as the duration of the inflammatory response is of relevance for the development of AKI. In experimental human endotoxemia, the intensity is limited (as no increase in tubular injury markers was found) and the duration is short-lived (11, 29). Therefore, this model of systemic inflammation in humans is too mild to induce AKI and to decrease GFR, but nevertheless, the correlation of these inflammatory mediators with the increase in GFR is very clear. This can indicate that the inflammation-induced renal response may orchestrate the pathophysiology of augmented renal function observed early in a subgroup of patients with sepsis (4, 30).

A strength of this study is the use of a gold standard method to measure GFR. De discrepancy between the correlations with inflammation between the “true” iohexol-derived GFR and the creatinine clearance-derived GFR advocates the use of accurate methods in studies that test mechanistical or pathophysiological risen hypotheses. However, methods using intravenously administered exogenous compounds such as iohexol are labor-intensive and have higher costs compared to creatinine-based methods. Another strength of this study is the standardized experimental translational design which limits confounders that influence the kidney, hemodynamics or the immune response; the healthy volunteers had a normal kidney function and the standardized LPS-dose and schedule elicits a reproducible and controlled inflammatory response (11). The absence of cardiac output measurements and the possibility to correct for changes in cardiac output is a limitation of the study. It is known that experimental human endotoxemia results in a hyperdynamic circulation with a high cardiac output (31). In septic patients a high cardiac output is an important and independent predictor of increased renal blood flow (RBF) (7). Nevertheless, we are able to conclude that the increase in GFR during experimental human endotoxemia is not dependent of perfusion pressure, as it was observed while blood pressure was significantly lower compared to baseline. In addition, an increase in cardiac output and RBF does not always result in an augmentation of the GFR. For example, concurrent vasodilatation during systemic inflammation may result in a lower glomerular capillary pressure and a low GFR (32). The correlations of the cytokines with the increase in the measured GFR we demonstrated suggest that systemic inflammation is an important determinant of the GFR, even when corrected for blood pressure.

In conclusion, in a highly standardized and controlled experimental endotoxemia study in healthy volunteers, the systemic immune response is significantly associated with the increase in GFR measured using the gold standard iohexol plasma clearance method, independent of hemodynamic effects. This correlation between the inflammatory response and an increase in GFR may explain the augmented GFR sometimes observed early in sepsis patients.

## Data Availability Statement

The raw data supporting the conclusions of this article will be made available by the authors on reasonable request, without undue reservation.

## Ethics Statement

The studies involving human participants were reviewed and approved by CMO Arnhem-Nijmegen. The participants provided their written informed consent to participate in this study.

## Author Contributions

PP, LE, MK, and RG designed the study. RG and GL conducted the study. RB, MS, and PP analyzed the data and drafted the manuscript. All authors revised the manuscript.

## Conflict of Interest

The authors declare that the research was conducted in the absence of any commercial or financial relationships that could be construed as a potential conflict of interest.
