# Supplementary material for: Endotoxemia-Induced Release of Pro-inflammatory Mediators Are Associated With Increased Glomerular Filtration Rate in Humans in vivo
Source: Front Med (Lausanne). 2020 Nov 5;7:559671. doi: 10.3389/fmed.2020.559671 (PMC7674961; doi:10.3389/fmed.2020.559671)
Supplement: Supplementary file 1 [file Data_Sheet_1.DOCX]

# Supplementary Material

Table 1. Correlation between inflammatory markers and GFR

|  | GFR_iohexol_ |  | GFR_ECC_ |  |
| --- | --- | --- | --- | --- |
|  | *R^2^* | *P* value | *R^2^* | *P* value |
| TNF-α | 0.33 | 0.0509 | 0.09 | 0.33 |
| IL-6 | 0.66 | 0.001*** | 0.09 | 0.36 |
| IL-10 | 0.03 | 0.62 | 0.0001 | 0.98 |
| IL-8 | 0.51 | 0.009*** | 0.01 | 0.74 |
| IL-1RA | 0.28 | 0.08 | 0.05 | 0.50 |
| _log_IL-12 | 0.009 | 0.77 | 0.04 | 0.55 |
| _log_MCP-1 | 0.38 | 0.03*** | 0.07 | 0.40 |
| MIP-1α | 0.005 | 0.82 | 0.03 | 0.61 |
| MIP-1β | 0.10 | 0.33 | 0.05 | 0.50 |
| ΔVCAM-1 | 0.37 | 0.04*** | 0.23 | 0.11 |
| ΔICAM-1 | 0.09 | 0.34 | 0.09 | 0.35 |
| *GFR: glomerular filtration rate; ECC: endogenous creatinine clearance; Δ: delta; log: logarithmic transformed data was used for statistical testing. For statistical comparison Pearson’s correlation coefficient was used, with a significant p-value p<0.05 (*)* | | | | |

Table 2. Correlation between blood pressure and GFR

|  | | GFR_iohexol_ | | GFR_ECC_ | |
| --- | --- | --- | --- | --- | --- |
|  | | *R^2^* | *P* value | *R^2^* | *P* value |
| Systolic Blood Pressure | | | | | |
|  | Peak | -0.02 | 0.95 | 0.11 | 0.74 |
|  | Nadir | -0.31 | 0.33 | -0.23 | 0.47 |
| Diastolic Blood Pressure | | | | | |
|  | Peak | 0.37 | 0.24 | 0.52 | 0.09 |
|  | Nadir | 0.13 | 0.69 | 0.06 | 0.86 |
| Mean Arterial Pressure | | | | | |
|  | Peak | 0.19 | 0.55 | 0.35 | 0.27 |
|  | Nadir | -0.09 | 0.77 | -0.10 | 0.76 |
| *GFR: glomerular filtration rate; ECC: endogenous creatinine clearance. For statistical comparison Pearson’s correlation coefficient was used, with a significant p-value p<0.05 (*)* | | | | | |
